# Targeting COPA to Enhance Erdafitinib Sensitivity in FGFR‐Altered Bladder Cancer

**DOI:** 10.1002/advs.202413209

**Published:** 2025-03-20

**Authors:** Huayuan Zhao, Xincheng Gao, Yangkai Jiang, Yanchao Yu, Liang Wang, Jiayin Sun, Miao Wang, Xing Xiong, Chao Huang, Hui Zhang, Guosong Jiang

**Affiliations:** ^1^ Department of Urology Union Hospital Tongji Medical College Huazhong University of Science and Technology Wuhan 430022 China; ^2^ Department of Urology The Third Affiliated Hospital of Nanchang University or The First Hospital of Nanchang 128 Xiangshan North Road Nanchang 330008 China; ^3^ Institute of Urology The Affiliated Luohu Hospital of Shenzhen University Shenzhen University Shenzhen 518000 China

**Keywords:** bladder cancer, COPA, erdafitinib resistance, m6A

## Abstract

Fibroblast growth factor receptor (FGFR) family aberrations are common in urothelial cancer. The FGFR tyrosine kinase inhibitor erdafitinib has been approved for locally advanced or metastatic urothelial cancer with FGFR2/3 alterations. Despite the initial efficacy of erdafitinib, resistance cannot be avoided. The molecular mechanisms underlying erdafitinib resistance have not been well investigated. Here, genome‐wide CRISPR screen is performed and coatomer protein complex subunit α (COPA) is identified as a key target to enhance erdafitinib sensitivity. Functionally, the deficiency of COPA reduces the proliferation of FGFR‐altered bladder cancer cells upon erdafitinib treatment. Mechanistically, COPA knockout increases the degradation of leucine‐rich pentatricopeptide repeat containing (LRPPRC) protein, leading to reduced inhibitor of DNA binding 3 (ID3) mRNA stability in an m6A‐dependent manner. Collectively, these findings reveal a novel mechanism of erdafitinib resistance, providing a potential therapeutic target for FGFR‐altered bladder cancer.

## Graphical abstract

Coatomer protein complex subunit α facilitates the transport of the cargo protein LRPPRC across the Golgi complex and enhances its stability, thereby increasing LRPPRC expression; LRPPRC promotes the stability of ID3 mRNA in an m6A‐dependent manner, thereby decreasing the transcriptional activity of p16 and p21, which leads to increased erdafitinib resistance in FGFR‐altered bladder cancer.

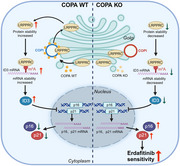

## 1 Introduction

Bladder cancer is one of the most common cancers in the world and the most costly cancer to treat on a per‐patient basis.^[^ ^1^ ^]^ Fibroblast growth factor receptors (FGFRs), a family of receptor tyrosine kinases (RTKs), regulate a variety of cellular processes. FGFR aberrations are frequently observed in a wide variety of cancers, most commonly in urothelial cancer.^[^ ^2^, ^3^ ^]^ The FDA granted accelerated approval to erdafitinib, an oral tyrosine kinase inhibitor (TKI) against all four members of the FGFR family (FGFR1‐4),^[^ ^4^ ^]^ for patients with FGFR2/3‐altered urothelial cancer in 2019.^[^ ^5^, ^6^ ^]^ Although erdafitinib treatment has shown positive clinical data in FGFR‐driven bladder cancer, objective responses were only observed in the minority of patients, with virtually all patients eventually experiencing disease progression, as both primary and acquired drug resistance limits the efficacy of erdafitinib treatment.^[^ ^7^ ^]^ Unfortunately, the mechanisms underlying erdafitinib resistance are complex and largely undefined. Therefore, further studies on the molecular mechanisms of erdafitinib resistance may shed light on the identification of novel targets to enhance erdafitinib sensitivity and therapeutic effect.

Coatomer protein complex subunit α (COPA) is a component of the coatomer protein complex I (COPI), which is involved in the transport of cargo proteins between the Golgi and the endoplasmic reticulum.^[^ ^8^ ^]^ Previous studies have shown that mutations in the COPA gene impair its transport function, which leads to abnormal trafficking of STING, causing a type I interferon‐mediated immune dysregulatory disease known as COPA syndrome.^[^ ^9^, ^10^, ^11^ ^]^ The WD40 domain of COPA contributes to the recruitment of cargo proteins into COPI vesicles during protein trafficking.^[^ ^12^ ^]^ Although COPA has also been reported to be associated with the regulation of cancer cell proliferation, migration, and invasion,^[^ ^13^, ^14^, ^15^ ^]^ its specific role and mechanism in regulating bladder cancer progression and sensitivity in targeted therapy remain unclear.

N6‐methyladenosine (m6A) is the most abundant internal modification of mRNA in eukaryotes.^[^ ^16^, ^17^ ^]^ Our recent studies have shown that increased m6A modification of TNFAIP3 mRNA could inhibit apoptosis of bladder cancer cells and decrease chemosensitivity to CDDP.^[^ ^18^ ^]^ The effects of m6A on mRNA can be mediated by an extended list of m6A readers.^[^ ^19^ ^]^ Leucine‐rich pentatricopeptide repeat containing (LRPPRC), an RNA‐binding protein, has recently been identified as a new m6A reader.^[^ ^20^, ^21^ ^]^ It has been reported that LRPPRC stabilizes the mRNA of IL‐11, CCND, and PLAU to promote metastasis and proliferation in bladder cancer.^[^ ^22^ ^]^ Nevertheless, the regulatory functions of LRPPRC as an m6A reader in targeted therapies of bladder cancer are yet to be revealed.

In this study, in order to find novel targets to enhance the sensitivity of erdafitinib therapy and explore the molecular mechanism underlying erdafitinib resistance, we performed genome‐wide CRISPR screen and found that sgRNAs of COPA were significantly absent in erdafitinib‐treated bladder cancer cells. The knockout of COPA enhanced the sensitivity of FGFR‐altered bladder cancer cells to erdafitinib, both in vivo and in vitro. Subsequent studies showed that COPA could interact with LRPPRC, and COPA knockout results in the accumulation of LRPPRC in the Golgi complex, as well as accelerated degradation. LRPPRC, as an m6A reader, promoted the stability of inhibitor of DNA binding 3 (ID3) mRNA in an m6A‐dependent manner, thereby decreasing the transcriptional activity of p16 and p21. Overall, our study highlights the role of COPA in regulating erdafitinib resistance, and provides a promising therapeutic target to enhance the sensitivity of erdafitinib treatment in FGFR‐altered bladder cancer.

## 2 Results

### 2.1 Absence of COPA Sensitized Bladder Cancer Cells to Erdafitinib Treatment

**Figure 1:**
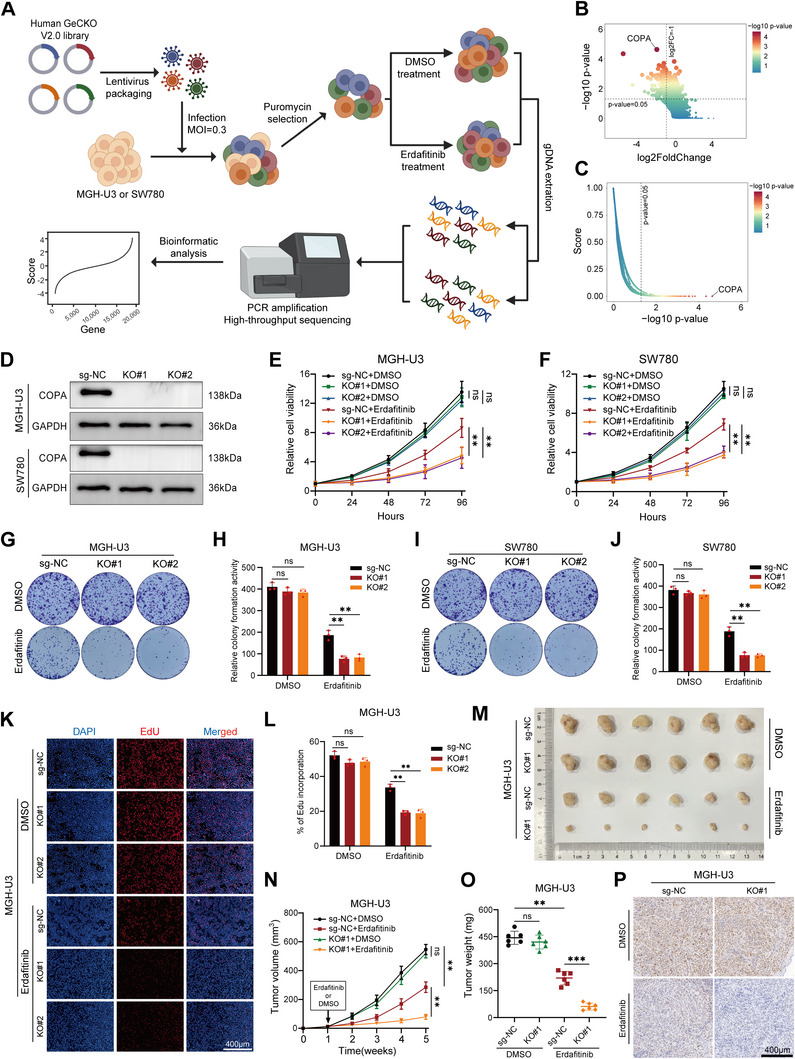
Absence of COPA sensitized bladder cancer cells to erdafitinib treatment. A) Schematic diagram illustrated the workflow of genome‐wide CRISPR/Cas9 knockout library scree on bladder cancer cells. B) Volcano plots showing the results of genome‐wide CRISPR/Cas9 negative screen. C) COPA was identified as the most significant gene in the negative screen as indicated. D) The efficiency of COPA knockout in MGH‐U3 and SW780 cells was detected by western blotting. GAPDH was used as internal control. E,F) CCK‐8 assay revealed the cell viability of MGH‐U3 and SW780 cells (before and after COPA knockout) treated with DMSO or erdafitinib (*n =* 3). G–J) Colony formation assay was performed in MGH‐U3 and SW780 cells (before and after COPA knockout) treated with DMSO or erdafitinib (*n =* 3). K,L) EdU assay showed the proliferation of sg‐NC and COPA‐KO MGH‐U3 cells treated with DMSO or erdafitinib (*n =* 3). Scale bar, 400 µm. M–O) Representative (M), in vivo growth curve (N), and weight at the endpoints (O) of xenograft tumors formed by subcutaneous injection of sg‐NC and COPA‐KO MGH‐U3 cells into the right flanks of nude mice treated with DMSO or erdafitinib (20 mg kg^−1^) (5 × 10^6^ cells per mouse; *n =* 6 for each group). **(P)** IHC staining of Ki67 on sg‐NC and COPA‐KO MGH‐U3 xenografts treated as in (N). Scale bar, 400 µm. Data are represented as mean ± SD. ns indicates not significant. **P < 0.01; ***P < 0.001 (Student t test).

To identify genes whose absence increases the sensitivity of bladder cells to erdafitinib, we performed genome‐wide CRISPR/Cas9 knockout library screen on MGH‐U3 (Y373C mutation) and SW780 (FGFR3‐BAIAP2L1 fusion) bladder cancer cell lines, both known to be FGFR3‐dependent^[^ ^23^, ^24^ ^]^ (**Figure** **1**A). From this CRISPR/Cas9 knockout library screen, cells carrying sgRNAs that target 178 genes were negatively selected (P < 0.05 and log2FC < ‐1) in the erdafitinib‐treated cells compared to the DMSO control, indicating that these genes may be potential targets for enhancing erdafitinib sensitivity (Figure 1B; Table S2, Supporting Information). Among the list of genes, COPA was identified as the most negatively selected gene upon erdafitinib treatment (Figure 1C). Meanwhile, we analyzed the RNA‐seq data from the EGA database (EGAD00001011063) of all 10 urothelial cancer patients treated with erdafitinib,^[^ ^7^ ^]^ and the sample from the only patient with progressive disease was detected the highest expression level of COPA, while the other nine patients were all observed with effective disease control (Table S3, Supporting Information). To further validate the results from our genome‐wide CRISPR screen, we generated COPA stable knockout (KO) subclones by infecting MGH‐U3 and SW780 cells with two independent sgRNAs. Two clones from each cell line that exhibited the most effective knockout effect in western blotting were ultimately selected for further analysis (Figure 1D). Subsequent studies demonstrated that COPA‐KO exhibited no statistically significant effect on bladder cancer cell proliferation. In contrast, knockout of COPA effectively impeded cell viability, colony formation ability, and cell proliferation of bladder cancer cells upon erdafitinib treatment (Figure 1E–L; Figure S1A,B, Supporting Information), but had no effect on apoptosis (Figure S1C–F, Supporting Information). To further investigate the regulation of COPA on erdafitinib sensitivity in vivo, COPA‐KO and sg‐NC cells were subcutaneously injected into nude mice. One week later, the mice were intraperitoneally injected with DMSO or erdafitinib daily for 4 weeks. We observed that the COPA knockout had no statistically significant effect on subcutaneous xenografts, while it markedly enhanced the erdafitinib‐induced inhibition in the growth of subcutaneous xenograft tumors (Figure 1M‐O; Figure S1G–I, Supporting Information). Meanwhile, the expression level of Ki‐67 in each group was consistent with the rate of tumor proliferation (Figure 1P; Figure S1J, Supporting Information). In addition, we aimed to investigate whether COPA is widely involved in regulating the sensitivity of tumor cells to various TKIs. Erdafitinib is the only FDA‐approved TKI for the treatment of FGFR‐altered bladder cancer and has not been approved for the targeted therapy of other cancers. Next, we knocked out COPA in 786‐O renal cancer cells (Figure S1K, Supporting Information), and found that COPA knockout could not enhance the sensitivity of 786‐O cells to sunitinib, a multitargeted TKI and standard first‐line treatment for patients with advanced renal cell carcinoma (Figure S1L‐N, Supporting Information). Therefore, we speculated that the regulation of COPA on TKI sensitivity may be specific in erdafitinib treatment of bladder cancer. Altogether, the above findings suggested that absence of COPA sensitized FGFR‐altered bladder cancer cells to erdafitinib treatment.

### 2.2 COPA Interacts with LRPPRC Protein

**Figure 2:**
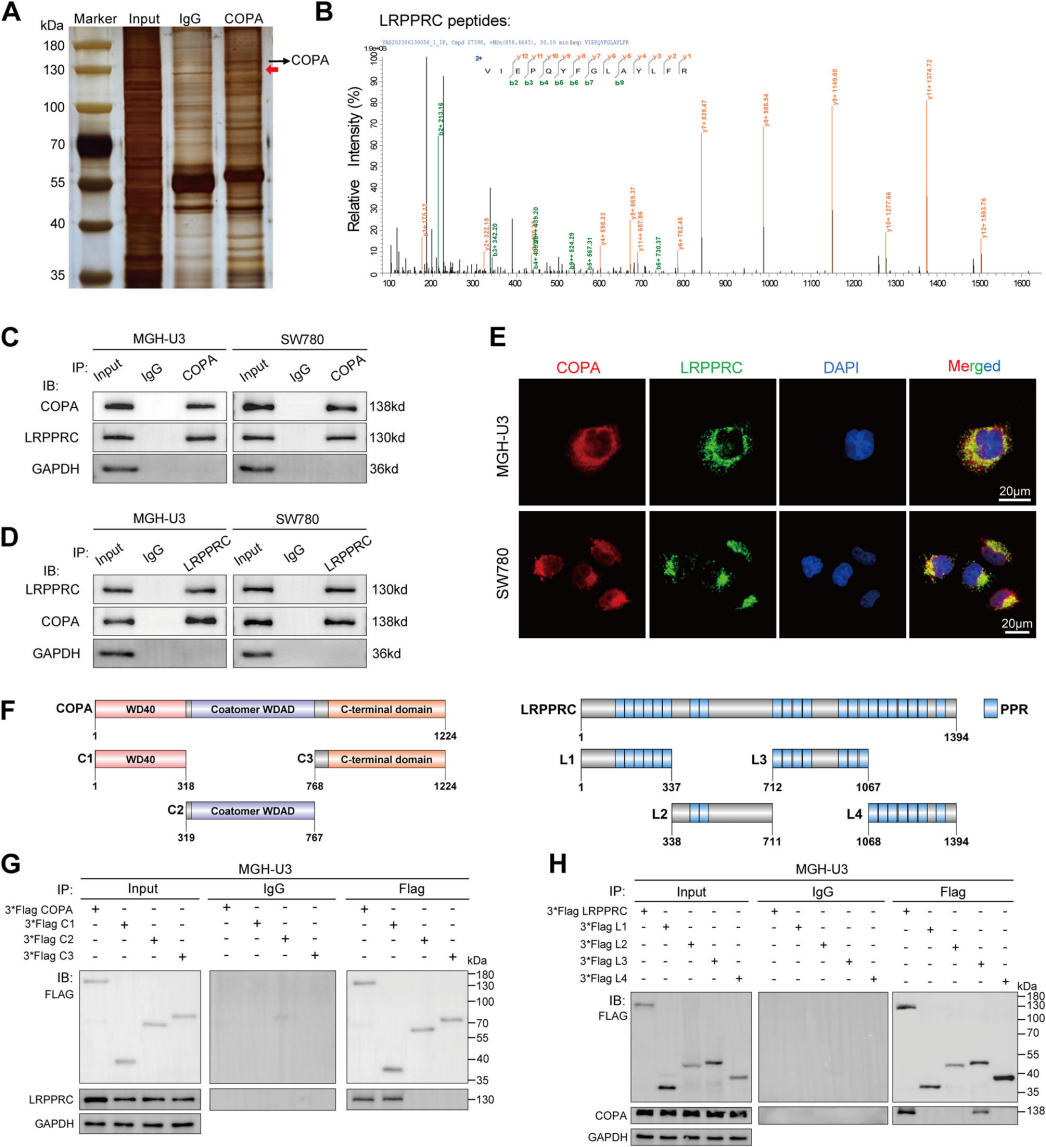
COPA interacts with LRPPRC protein. A) Silver staining showed the proteins pulled down by COPA from the lysates of MGH‐U3 cells. Red arrow indicated the major differential band precipitated. B) Mass spectrometry assay showed the identified LRPPRC peptides pulled down by COPA in MGH‐U3 lysates. C) Co‐IP assay using antibody specific for COPA showed the interaction between COPA and LRPPRC in MGH‐U3 and SW780 cells. The precipitate was subjected to western blotting with the antibodies against COPA, LRPPRC, and GAPDH. D) Co‐IP assay using antibody specific for LRPPRC showed the interaction between COPA and LRPPRC in MGH‐U3 and SW780 cells. The precipitate was subjected to western blotting with the antibodies against LRPPRC, COPA, and GAPDH. E) Immunofluorescence staining assay showed the co‐localization of LRPPRC (green) and COPA (red) in the cytoplasm of MGH‐U3 and SW780 cells, with nuclei staining with DAPI (blue). Scale bar, 20 µm. F) Schematic diagram revealed the functional domains of LRPPRC and COPA from previous reports and SMART database (https://smart.embl.de/), and the domains of LRPRC and COPA truncations. G) Co‐IP assay using antibody specific for Flag showed the interaction between LRPPRC and full‐length or truncations of Flag‐tagged recombinant COPA in MGH‐U3 cells. The precipitate was subjected to western blotting with the antibodies against Flag, LRPPRC, and GAPDH. H) Co‐IP assay using antibody specific for Flag showed the interaction between COPA and full‐length or truncations of Flag‐tagged recombinant LRPPRC in MGH‐U3 cells. The precipitate was subjected to western blotting with the antibodies against Flag, COPA, and GAPDH.

Immunofluorescence assay and nuclear/cytoplasmic protein extraction assay indicated that COPA was mainly located in the cytoplasm of MGH‐U3 and SW780 cells (Figure S2A,B, Supporting Information). In light of the function of COPA as a subunit of COPI vesicles, which are responsible for recognizing and binding cargo proteins and regulating protein transport, we used co‐immunoprecipitation (Co‐IP) assays to identify downstream target proteins. Potential interacting proteins were identified by silver staining and mass spectrometry analysis (**Figures** **2**A,B; S2C, Supporting Information). Other subunits of COPI, including COPG1, COPB1, and COPB2, were found among the identified proteins, thereby corroborating the reliability of the experimental results (Table S4, Supporting Information). Among the identified proteins with unique peptides ≥ 10, the fold change of LRPPRC was ranked first. The interaction between COPA and LRPPRC was further confirmed through Co‐IP assays (Figure 2C,D). Likewise, COPA and LRPPRC were colocalized in the cytoplasm of bladder cancer cells (Figure 2E). To describe the structural determinants of the interaction between COPA and LRPPRC, we subdivided the functional domains of COPA and LRPPRC based on the SMART database and previous literature references^[^ ^25^ ^]^ (Figure 2F). Subsequent studies demonstrated that the WD40 domain (aa1‐318) of COPA and the PPR motifs 9–15 (aa712‐1067) of LRPPRC mediated the interaction (Figure 2G,H). In summary, these results proposed that COPA and LRPPRC formed a protein‐protein complex through the WD40 domain of COPA and PPR motifs 9–15 of the 22 PPR motifs of LRPPRC.

### 2.3 COPA Depletion Enhances Erdafitinib Sensitivity via LRPPRC in Bladder Cancer Cells

**Figure 3:**
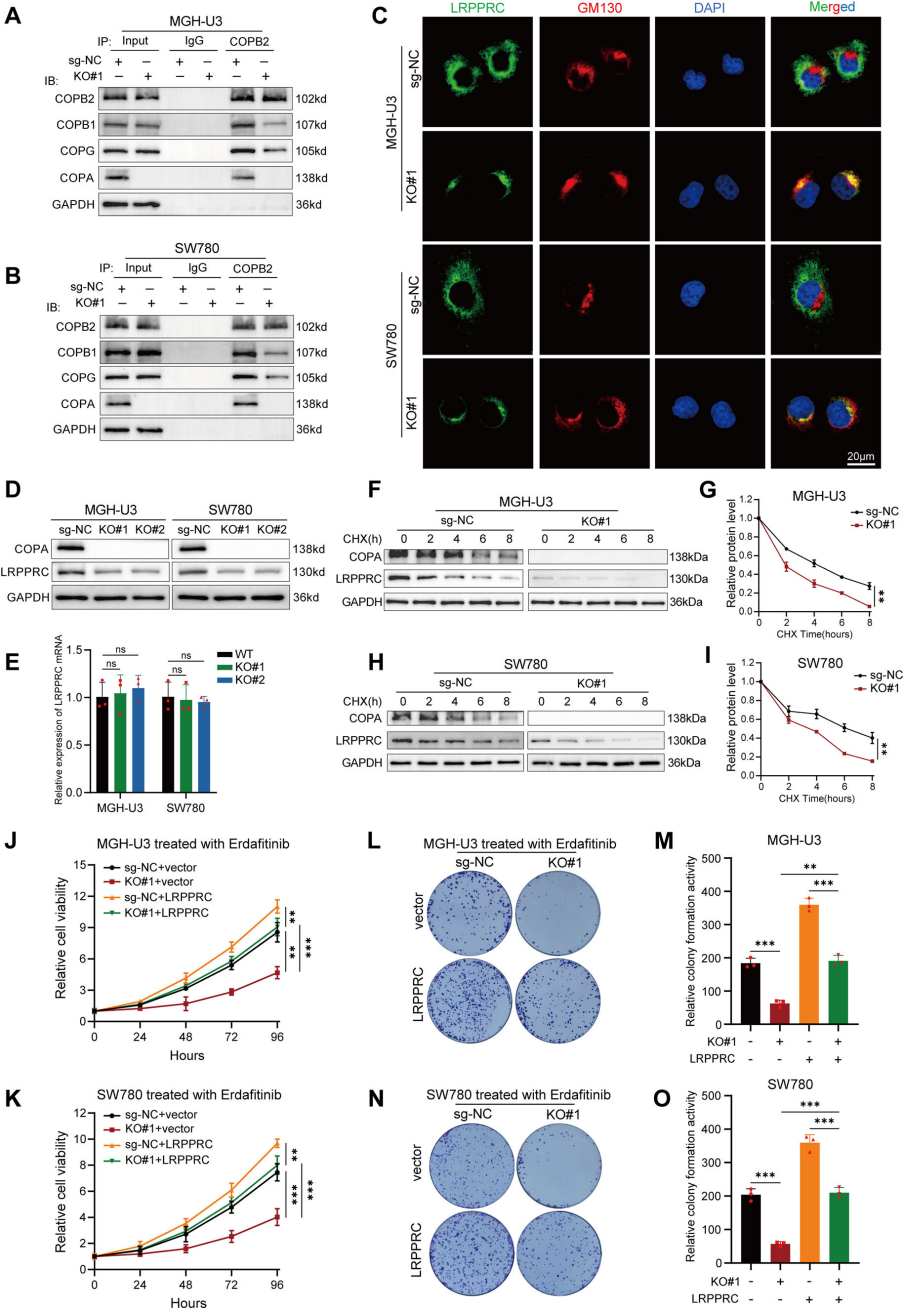
COPA depletion enhances erdafitinib sensitivity via LRPPRC. A,B) Co‐IP assay using antibody specific for COPB2 showed the interaction between COPB2 and other residual COPI subunits in MGH‐U3 and SW780 cells. The precipitate was subjected to western blotting with the indicated antibodies. C) Immunofluorescence staining assay showed the subcellular localization of LRPPRC (green) and GM130 (red) in MGH‐U3 and SW780 cells, with nuclei staining with DAPI (blue). Scale bar, 20 µm. D,E) The expression of LRPPRC in MGH‐U3 and SW780 cells before and after COPA knockout was detected by western blotting (A) and qRT‐PCR (B) (*n =* 3). GAPDH was used as internal control. F,G) Western blot of LRPPRC stability after treatment with CHX (50 µg/mL) for the indicated time in sg‐NC and COPA‐KO MGH‐U3 cells. GAPDH was used as internal control (*n =* 3). (H, I) Western blot of LRPPRC stability after treatment with CHX (50 µg/mL) for the indicated time in sg‐NC and COPA‐KO SW780 cells. GAPDH was used as internal control (*n =* 3). J,K) CCK‐8 assay revealed the cell viability of MGH‐U3 and SW780 cells (before and after COPA knockout) transfected with vector or LRPPRC treated with erdafitinib (*n =* 3). (L‐O) Colony formation assay was performed in MGH‐U3 and SW780 cells (before and after COPA knockout) transfected with vector or LRPPRC treated with erdafitinib (*n =* 3). Data are represented as mean ± SD. ns indicates not significant. **P < 0.01; ***P < 0.001 (Student t test).

COPI is a stable complex that is integrally recruited from the cytosol to the Golgi membrane,^[^ ^26^ ^]^ and it is assembled from trimeric COPA, COPB2, COPE subcomplex, and the tetrameric COPB1, COPG, COPD, COPZ subcomplex.^[^ ^27^ ^]^ It was previously reported that abnormality of COPI subunits leads to impaired COPI assembly and the disrupted function of transport.^[^ ^28^, ^29^, ^30^ ^]^ Therefore, we hypothesized that COPA depletion might interfere with the transport of cargo protein LRPPRC across the Golgi complex during biosynthesis. First, we performed Co‐IP assay to confirm endogenous binding of COPA, COPB2, COPB1, and COPG in bladder cancer cells (Figure S3A, Supporting Information). Next, we found that COPA depletion almost had no influence on the expression levels of residual COPI subunits (Figure S3B, Supporting Information). Importantly, the absence of COPA resulted in a significant reduction in the interaction between other COPI subunits (**Figure** **3**A,B), indicating impaired COPI assembly. Moreover, COPA depletion resulted in the accumulation of LRPPRC in the Golgi complex (Figure 3C), suggesting that the transport function of COPI may be impaired. Subsequently, we tried to investigate the effect of COPA on the expression of LRPPRC protein. Our findings indicated that knockout of COPA significantly down‐regulated the protein expression of LRPPRC (Figure 3D), with no statistical effect on the mRNA level of LRPPRC (Figure 3E). The Golgi is the main factory for post‐translational modifications and protein maturation, which are essential for protein stability.^[^ ^31^, ^32^ ^]^ To determine whether COPA knockout could promote LRPPRC degradation, we treated COPA‐KO and sg‐NC cells with protein synthesis inhibitor cycloheximide. Of note, the results confirmed that COPA knockout caused a significant decrease in the half‐life of LRPPRC (Figure 3F–I). Importantly, the overexpression of LRPPRC reversed the suppressive cell viability caused by COPA knockout (Figure 3J,K). Moreover, the reduced colony formation ability in COPA‐KO cells was also abolished by LRPPRC overexpression (Figure 3L–O). These findings confirmed that COPA knockout can promote the degradation of LRPPRC protein and downregulate its expression, resulting in increased erdafitinib sensitivity in bladder cancer cells.

### 2.4 COPA Regulates the Expression of ID3 through LRPPRC

**Figure 4:**
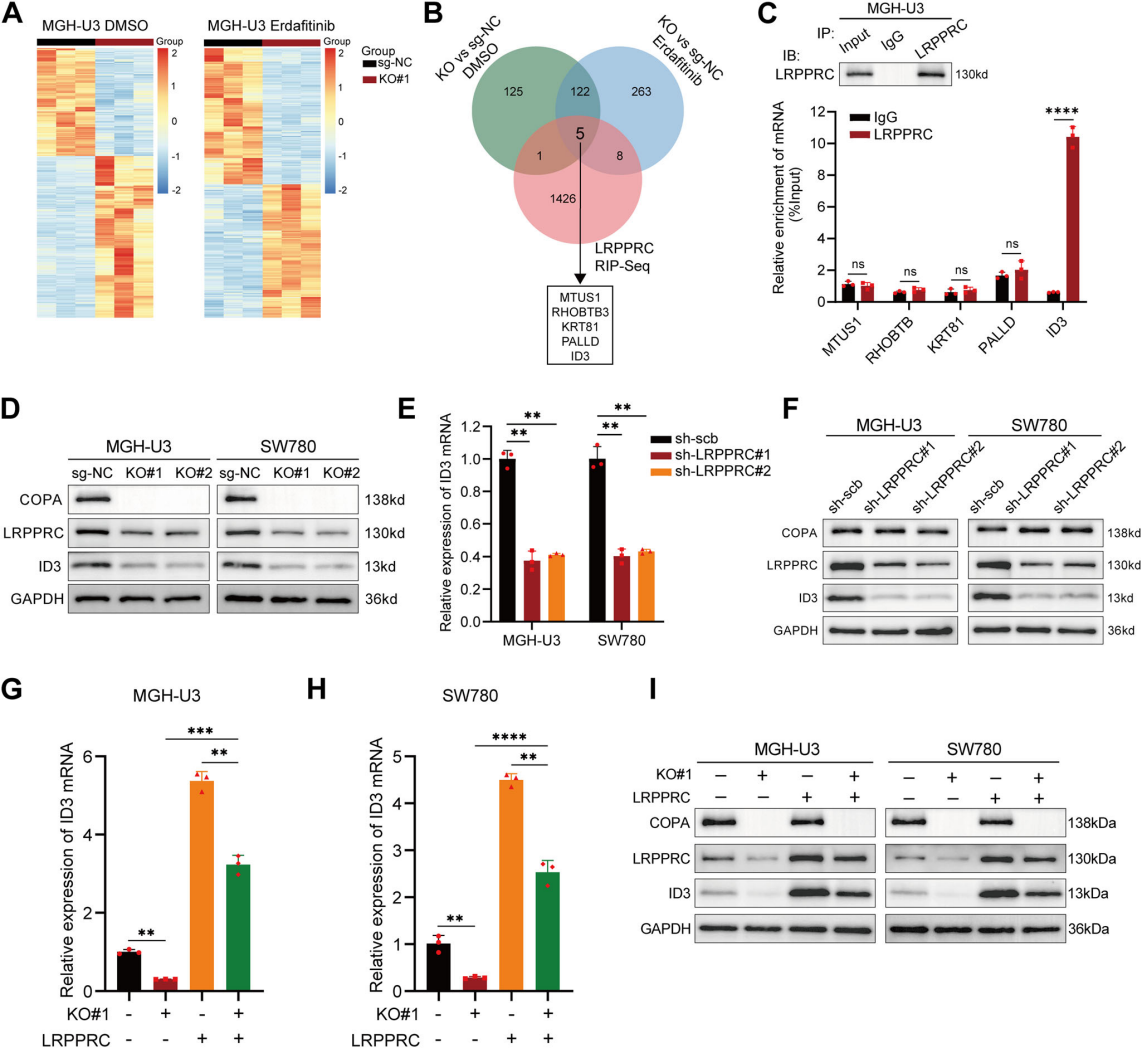
ID3 is the target of LRPPRC in the regulation of erdafitinib sensitivity. A) Heatmap depicted the differentially expressed mRNA between sg‐NC and COPA‐KO cells treated with DMSO (left) or erdafitinib (right) for 96 h (p < 0.05). Each group contained three independent replicates respectively. B) Venn diagram showed the downregulated genes detected by RNA‐seq upon COPA knockout in MGH‐U3 cells and overlapping analysis with LRPPRC RIP‐Seq results from the GEO database. C) RIP assays in MGH‐U3 cells using LRPPRC and IgG antibody. The precipitate was subjected to western blotting with the antibody against LRPPRC. The LRPPRC‐enriched mRNAs relative to the IgG‐enriched value were calculated by qRT‐PCR (*n =* 3). D) Western blotting with the indicated antibodies in MGH‐U3 and SW780 cells before and after COPA knockout. GAPDH was used as internal control. E) The expression of ID3 was detected by qRT‐PCR in MGH‐U3 and SW780 cells transfected with scramble, sh‐LRPPRC#1 or sh‐LRPPRC#2 (*n =* 3). F) Western blotting with the indicated antibodies in MGH‐U3 and SW780 cells transfected with scramble, sh‐LRPPRC#1, or sh‐LRPPRC#2. GAPDH was used as internal control. G–I) The expression of ID3 in MGH‐U3 and SW780 cells (before and after COPA knockout) transfected with vector or LRPPRC was detected by qRT‐PCR (G, H) (*n =* 3) and western blotting (I). GAPDH was used as internal control. Data are represented as mean ± SD. ns indicates not significant. **P < 0.01; ***P < 0.001, ****P < 0.0001 (Student t test).

It has been demonstrated that LRPPRC, as an RNA‐binding protein, can enhance mRNA stability.^[^ ^20^, ^22^, ^33^, ^34^ ^]^ To identify the target genes and downstream signaling pathways, transcriptome analysis was performed in COPA‐KO and sg‐NC cells upon either DMSO or erdafitinib treatment (**Figure** **4**A). It was revealed that 253 RNAs were down‐regulated in COPA‐KO cells upon DMSO treatment, while 398 RNAs were down‐regulated in COPA‐KO cells upon erdafitinib treatment (Table S5, Supporting Information). Comprehensive analysis of LRPPRC RIP‐seq results from the GEO datasets (GSE204923) with our RNA‐seq results indicated that five targets might be regulated by LRPPRC (Figure 4B), including MTUS1, RHOBTB3, KRT81, PALLD, and ID3. Next, we used RIP‐qPCR to validate whether LRPPRC could bind to these candidate mRNAs. As shown in Figure 4C and Figure S4A (Supporting Information), only ID3 mRNA was significantly enriched in LRPPRC group compared with IgG group. Furthermore, consistent with our RNA‐seq results, knockout of COPA markedly decreased ID3 expression (Figure S4B, Supporting Information, Figure 4D). Therefore, we speculated that COPA knockout reduced ID3 mRNA stability by decreasing the protein expression of LRPPRC. Subsequently, we confirmed that LRPPRC knockdown could downregulate the expression of ID3 (Figure S3C, Supporting Information; Figure 4E,F). In addition, overexpression of LRPPRC could reverse the decline in ID3 expression induced by COPA knockout (Figure 4G–I). These findings suggested that COPA and its downstream protein LRPPRC regulated the expression of ID3, which was the target of LRPPRC.

### 2.5 LRPPRC Promotes ID3 mRNA Stability in an m6A‐Dependent Manner

**Figure 5:**
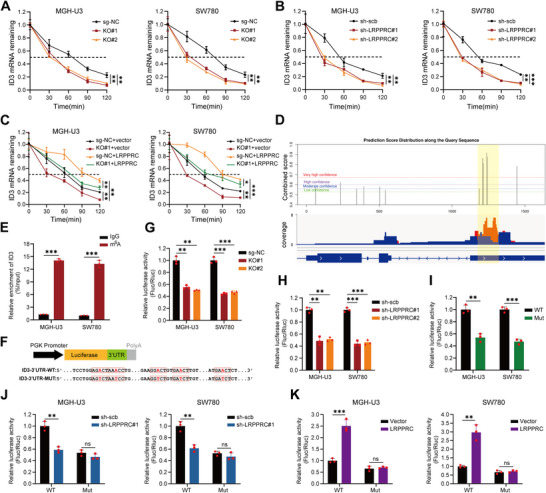
LRPPRC promotes ID3 mRNA stability in an m6A‐dependent manner. A) The relative remaining levels of ID3 mRNA in MGH‐U3 and SW780 cells before and after COPA knockout was analyzed by qRT‐PCR after treatment with actinomycin D (5 µg µL^−1^) at the indicated time points (*n =* 3). B) The relative remaining levels of ID3 mRNA in MGH‐U3 and SW780 cells transfected with scramble, sh‐LRPPRC#1 or sh‐LRPPRC#2 was analyzed by qRT‐PCR after treatment with actinomycin D (5 µg µL^−1^) at the indicated time points (*n =* 3). C) The relative remaining levels of ID3 mRNA in MGH‐U3 and SW780 cells (before and after COPA knockout) transfected with vector or LRPPRC was analyzed by qRT‐PCR after treatment with actinomycin D (5 µg µL^−1^) at the indicated time points (*n =* 3). D) The potential m6A sites of ID3 predicted by SRAMP software were combined and co‐localized with MeRIP‐seq results of bladder cancer in GEO datasets. E) MeRIP assays in MGH‐U3 and SW780 cells using m6A and IgG antibody. The m6A‐enriched ID3 mRNA relative to the IgG‐enriched value was calculated by qRT‐PCR (*n =* 3). F) Schematic diagram of ID3‐3′UTR‐WT and ID3‐3′UTR‐MUT luciferase reporters. G) The relative luciferase activity of ID3‐3′UTR‐WT in MGH‐U3 and SW780 cells before and after COPA knockout (*n =* 3). H) The relative luciferase activity of ID3‐3′UTR‐WT in MGH‐U3 and SW780 cells transfected with scramble, sh‐LRPPRC#1 or sh‐LRPPRC#2 (*n =* 3). I) The relative luciferase activity in MGH‐U3 and SW780 cells transfected with ID3‐3′UTR‐WT or ID3‐3′UTR ‐MUT luciferase reporter plasmids (*n =* 3). J) The relative luciferase activity of ID3‐3′UTR‐WT or ID3‐3′UTR‐MUT reporters in MGH‐U3 and SW780 cells transfected with scramble or sh‐LRPPRC#1 (*n =* 3). K) The relative luciferase activity of ID3‐3′UTR‐WT or ID3‐3′UTR‐MUT reporters in MGH‐U3 and SW780 cells transfected with vector or LRPPRC (*n =* 3). Data are represented as mean ± SD. ns indicates not significant. **P < 0.01; ***P < 0.001 (Student t test).

Given the role of LRPPRC in enhancing mRNA stability, we explored the difference in ID3 mRNA stability in sg‐NC and COPA‐KO cells by RNA degradation assay. As expected, COPA knockout significantly reduced ID3 mRNA stability (**Figure** **5**A). Likewise, LRPPRC knockdown led to a similar reduction in ID3 mRNA stability (Figure 5B). Furthermore, overexpression of LRPPRC could reverse the decline in ID3 mRNA stability caused by COPA knockout (Figure 5C). Recent studies have demonstrated that LRPPRC functions as an m6A reader by binding to m6A‐methylated transcripts. Based on the SRAMP software analysis, we identified five very high‐confidence m6A sites in the 3′UTR region upon ID3 mRNA. Meanwhile, by analyzing the MeRIP‐seq results of bladder cancer in GEO datasets (GSE231836), we found an obvious m6A peak in the 3′UTR region of ID3 mRNA, which aligned with the sites predicted by SRAMP software (Figure 5D). MeRIP‐qPCR further revealed abundant m6A modifications on ID3 mRNA in bladder cancer cell (Figure 5E). We next inserted either the wild‐type or m6A‐site‐mutant 3′UTR sequence into the luciferase reporter vector for luciferase reporter assays (Figure 5F). We observed that COPA knockout reduced ID3 3′UTR luciferase activity (Figure 5G). Similar results were observed in LRPPRC‐knockdown cells (Figure 5H). Of note, mutations of the m6A sites resulted in reduced luciferase activity compared to the wild type (Figure 5I). Meanwhile, the impact of LRPPRC knockdown or overexpression on luciferase activity was blocked by mutations of the m6A sites (Figure 5J,K). Taken together, these findings suggested that LRPPRC recognized the 3′UTR region upon ID3 mRNA and stabilized ID3 mRNA in an m6A‐dependent manner.

### 2.6 COPA/LRPPRC/ID3 Pathway Modulates Erdafitinib Sensitivity via p16 and p21

**Figure 6:**
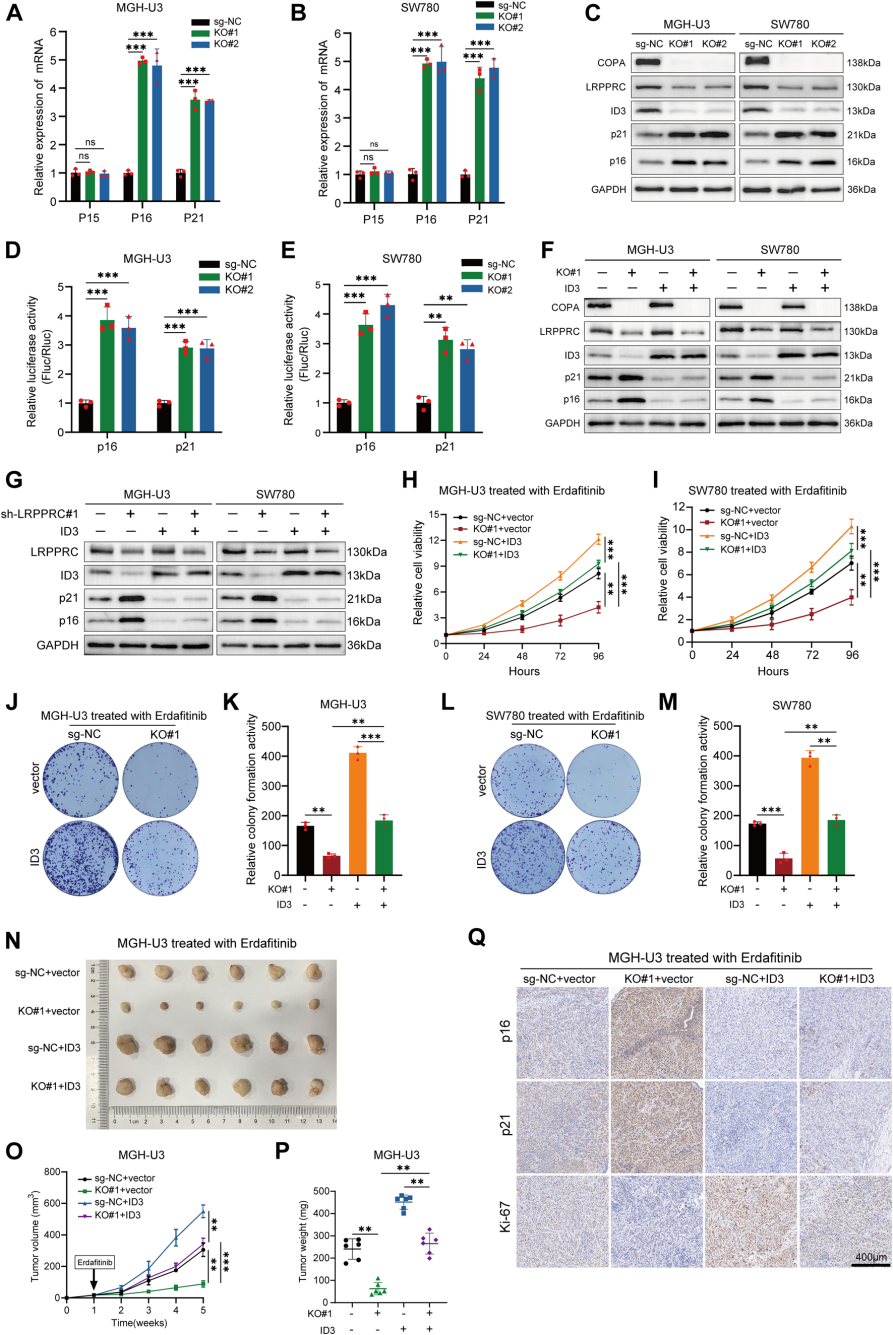
ID3 mediates the regulation of COPA on the transcriptional activity of p16 and p21, as well as on the sensitivity to erdafitinib. A,B) The expression of p15, p16, and p21 in MGH‐U3 (A) and SW780 (B) cells before and after COPA knockout was detected by qRT‐PCR (*n =* 3). (C) Western blotting with the indicated antibodies in MGH‐U3 and SW780 cells before and after COPA knockout. GAPDH was used as internal control. D,E) The relative luciferase activity of p16 and p21 promoter in MGH‐U3 (D) and SW780 (E) cells before and after COPA knockout (*n =* 3). F) Western blotting with the indicated antibodies in MGH‐U3 and SW780 cells (before and after COPA knockout) transfected with vector or ID3. GAPDH was used as internal control. G) Western blotting with the indicated antibodies in MGH‐U3 and SW780 cells transfected with scramble or sh‐LRPPRC#1, and those co‐transfected with vector or ID3. GAPDH was used as internal control.2. H,I) CCK‐8 assay revealed the cell viability of MGH‐U3 (H) and SW780 (I) cells (before and after COPA knockout) transfected with vector or ID3 treated with erdafitinib (*n =* 3). J–M) Colony formation assay was performed in MGH‐U3 and SW780 cells (before and after COPA knockout) transfected with vector or ID3 treated with erdafitinib (*n =* 3). N–P) Representative (N), in vivo growth curve (O), and weight at the endpoints (P) of xenograft tumors formed by subcutaneous injection of sg‐NC and COPA‐KO MGH‐U3 cells transfected with vector or ID3 into the right flanks of nude mice treated with erdafitinib (20 mg kg^−1^) (5 × 10^6^ cells per mouse; *n =* 6 for each group). (Q) IHC staining of Ki‐67, p16, and p21 on sg‐NC and COPA‐KO MGH‐U3 xenografts treated as in (O). Scale bar, 400 µm. Data are represented as mean ± SD. ns indicates not significant. **P < 0.01; ***P < 0.001 (Student t test).

It has been reported that ID3 contributes to cancer progression by antagonizing the transcriptional activation of cyclin‐dependent kinase inhibitors (CDKIs), such as p15, p16, and p21.^[^ ^35^, ^36^ ^]^ Hence, we explored whether ID3 regulates the expression of CDKIs in bladder cancer. As shown in the results, ID3 knockdown resulted in the activation of p16 and p21 promoters, as well as the up‐regulated expression of p16 and p21, while having no effect on p15 (Figure S5A–F, Supporting Information). To further elucidate the functional mechanisms underlying COPA, we detected the changes in CDKIs expression regulated by ID3 in sg‐NC and COPA‐KO cells. Notably, COPA knockout also significantly increased the expression and promoter luciferase activity of p16 and p21 (**Figure** **6**A–E). Likewise, in our RNA‐seq results, statistically significant increases in p16 and p21 expression were also detected (Figure S5G,H, Supporting Information). To confirm whether the activation of p16 and p21 transcription caused by COPA knockout was mediated by the reduction of ID3, we overexpressed ID3 in sg‐NC and COPA‐KO cells. As shown in Figure 6F and Figure S5I–L (Supporting Information), overexpression of ID3 rescued the increased expression of p16 and p21, as well as the promoter activation induced by COPA knockout. Furthermore, overexpression of ID3 also reversed the upregulation in p16 and p21 expression caused by LRPPRC knockdown (Figure 6G). Importantly, we found that overexpression of ID3 rescued the suppressive cell viability and colony formation ability in COPA‐KO cells upon erdafitinib treatment (Figure 6H–M). Consistently, overexpression of ID3 reversed the decrease in volume and weight of subcutaneous xenograft tumors of COPA‐KO cells (Figure 6N–P; Figure S5M–O, Supporting Information). Consistently, IHC analysis revealed that the decrease in Ki‐67 caused by COPA knockout was rescued by ID3 overexpression (Figure 6Q; Figure S5P, Supporting Information).

**Figure 7:**
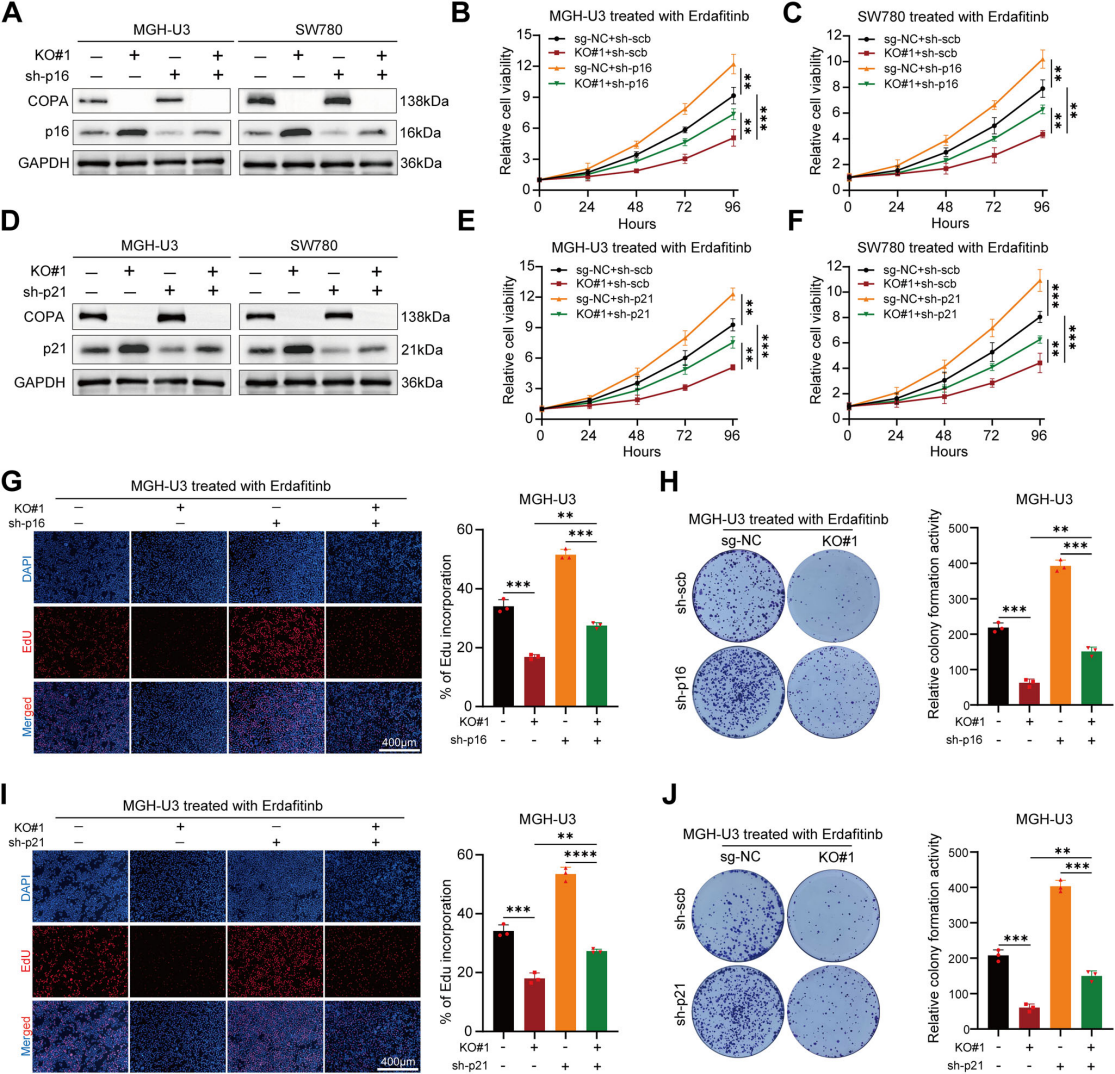
Knockdown of p16 or p21 partially rescued the up‐regulated sensitivity to erdafitinib induced by COPA knockout. A) Western blotting with the indicated antibodies in MGH‐U3 and SW780 cells (before and after COPA knockout) transfected with scramble or sh‐p16. GAPDH was used as internal control. B,C) CCK‐8 assay revealed the cell viability of MGH‐U3 (B) and SW780 (C) cells (before and after COPA knockout) transfected with scramble or sh‐p16 treated with erdafitinib (*n =* 3). D) Western blotting with the indicated antibodies in MGH‐U3 and SW780 cells (before and after COPA knockout) transfected with scramble or sh‐p21. GAPDH was used as internal control. E,F) CCK‐8 assay revealed the cell viability of MGH‐U3 (E) and SW780 (F) cells (before and after COPA knockout) transfected with scramble or sh‐p21 treated with erdafitinib (*n =* 3). G) EdU assay showed the proliferation of sg‐NC and COPA‐KO MGH‐U3 cells transfected with scramble or sh‐p16 treated with erdafitinib (*n =* 3). Scale bar, 400 µm. H) Colony formation assay was performed in sg‐NC and COPA‐KO MGH‐U3 cells transfected with scramble or sh‐p16 treated with erdafitinib (*n =* 3). I) EdU assay showed the proliferation of sg‐NC and COPA‐KO MGH‐U3 cells transfected with scramble or sh‐p21 treated with erdafitinib (*n =* 3). Scale bar, 400 µm. J) Colony formation assay was performed in sg‐NC and COPA‐KO MGH‐U3 cells transfected with scramble or sh‐p21 treated with erdafitinib (*n =* 3). Data are represented as mean ± SD. **P < 0.01; ***P < 0.001 (Student t test).

To confirm whether the effects of COPA on erdafitinib sensitivity were mediated via p16 and p21, we conducted a series of rescue experiments. The results showed that the knockdown of p16 and p21 partially abolished the decrease in cell viability induced by COPA knockout upon erdafitinib treatment (**Figure** **7**A–F). Additionally, the inhibition of cell proliferation and colony formation ability caused by COPA knockout upon erdafitinib treatment was also partially rescued by the knockdown of p16 and p21 (Figure 7G–J; Figure S6A–D, Supporting Information). Collectively, these results demonstrated that COPA/LRPPRC/ID3 pathway modulated erdafitinib sensitivity by regulating the expression of p16 and p21.

## 3 Discussion

Although erdafitinib has shown promising clinical outcomes in the treatment of FGFR‐driven bladder cancer, the inevitable development of therapeutic resistance limits the efficacy.^[^ ^37^ ^]^ Thus, further investigation is still required to elucidate the molecular mechanisms underlying erdafitinib resistance. It has been reported that FGFR kinase domain mutations and PIK3CA activation mutations contribute to erdafitinib acquired resistance in FGFR‐driven bladder cancers.^[^ ^7^ ^]^ Furthermore, paracrine activation of the NRG1/HER3 axis mediated by adipocyte precursors can also promote resistance to erdafitinib rapidly.^[^ ^38^ ^]^  In contrast, our work uncovers a novel intrinsic resistance mechanism. In this study, we performed genome‐wide CRISPR screen and identified COPA as a critical gene associated with erdafitinib sensitivity in FGFR‐driven bladder cancer. Moreover, we discovered that COPA knockout increased the degradation of LRPPRC protein, which resulted in reduced stability of ID3 mRNA in an m6A‐dependent manner. In conclusion, our study highlights a significant mechanism of erdafitinib resistance and suggests that COPA could serve as a promising target to enhance the sensitivity of erdafitinib treatment in FGFR‐altered bladder cancer.

COPA is a unit of COPI vesicles which contribute to protein trafficking. We found that COPA could interact with LRPPRC. Previous reports have shown that the WD40 domain of COPA targets cargo proteins into COPI vesicles,^[^ ^12^ ^]^ and our study confirmed that COPA also interacted with LRPPRC through the WD40 domain. COPI have been shown to play a central role in proteins transport between the Golgi and the endoplasmic reticulum,^[^ ^8^ ^]^ and is also involved in the anterograde transport of cargo proteins in the Golgi during protein biosynthesis.^[^ ^39^ ^]^ The Golgi serves as the major factory for post‐translational modifications and protein maturation, which is essential for protein stability.^[^ ^31^, ^32^ ^]^ Indeed, it has also been reported that block of COPI function prevents normal post‐translational modifications and maturation of cargo protein APP.^[^ ^40^, ^41^ ^]^ Here, our study demonstrated that the knockout of COPA impaired COPI assembly and lead to the accumulation of LRPPRC within the Golgi complex and its decreased stability. Therefore, we speculate that the loss of COPI function interferes with the normal transport of LRPPRC across the Golgi during protein biosynthesis, resulting in abnormal post‐translational modification and maturation, leading to decreased stability of LRPPRC. Although the specific mechanism still needs to be further clarified, these results provide new insights into the regulation of COPA on the stability of cargo protein.

Notably, LRPPRC has recently been identified as a reader for m6A modification sites.^[^ ^21^ ^]^ Subsequently, it has been shown that LRPPRC facilitates the progression of breast and liver cancer by enhancing mRNA stability, which was mediated by m6A modification.^[^ ^20^, ^42^ ^]^ However, the functions of LRPPRC as an m6A reader in bladder cancer remain poorly understood. In the current study, we found that ID3 was a novel direct downstream target of LRPPRC‐mediated regulation on mRNA stability in an m6A‐dependent manner. These results provide new evidence for LRPPRC as an m6A reader in regulating tumor progression during targeted therapy. Nevertheless, it is worth mentioning that although LRPPRC had an all‐helical structure and contained 22 PPR motifs,^[^ ^25^ ^]^ the specific functional PPR motifs that mediate binding to the m6A modification require further characterization.

ID3 is a helix‐loop‐helix (HLH) protein that can bind and sequester class I and II bHLH transcription factors in non‐DNA‐binding dimers, thereby negatively regulating gene expression.^[^ ^35^ ^]^ It has been reported that ID3 is extensively involved in the regulation of the transcriptional activity of CDKIs.^[^ ^36^, ^43^, ^44^ ^]^ Our results verified that LRPPRC‐m6A‐ID3 axis mediated erdafitinib resistance by inhibiting the transcriptional activity of p16 and p21 in bladder cancer. Hence, targeting this axis may offer a promising therapeutic strategy to overcome erdafitinib resistance.

In this study, we elaborate a working model of the COPA/LRPPRC/ID3 axis across different cellular compartments: (1) the COPA subunit of COPI interacts with the cargo protein LRPPRC and facilitates its anterograde transport through the Golgi complex during protein biosynthesis; (2) the mature LRPPRC protein leaves the Golgi and stabilizes ID3 mRNA in cytoplasm; and (3) the up‐regulated ID3 protein enters the nucleus to regulate the transcriptional activity of CDKIs.

In summary, the insights obtained from this study significantly enhance our understanding of the erdafitinib resistance in FGFR‐altered bladder cancer. We provide comprehensive evidence that the COPA/LRPPRC/ID3 pathway modulates erdafitinib sensitivity through p16 and p21. Remarkably, COPA may serve as a potential therapeutic target to improve the efficacy of erdafitinib therapy for FGFR‐driven bladder cancer patients.

## 4 Experimental Section

### Cell Culture and Treatment

Human bladder cancer cell lines MGH‐U3 and SW780 were purchased from American Type Culture Collection (ATCC, USA). The human renal cancer cell lines 786‐O (CL‐0010) was purchased from Procell Life Science & Technology (Wuhan, China). All the cell lines were cultured in RPMI‐1640 medium (Gibco, USA) supplemented with 10% FBS (Gibco, USA), 1% penicillin/streptomycin (Gibco, USA). Cells were cultured in an incubator at 37 °C with humidified atmosphere of 5% CO2. All bladder cancer cell lines were confirmed within 6 months before use by using a short tandem repeat profiling and were confirmed negative for Mycoplasma contamination. Erdafitinib (MCE, China) was solubilized in DMSO.

### Genome‐Wide CRISPR Library Screen

The Human GeCKO v2 CRISPR knockout pooled library was utilized to screen for genes driving erdafitinib resistance in bladder cancer cells.^[^ ^45^ ^]^ MGH‐U3 and SW780 cells were transduced with the GeCKO v2 library, which contains 122411 unique sgRNAs targeting 19052 human genes and 1864 miRNAs (6 sgRNAs per gene, 4 sgRNAs per miRNA, and 1000 non‐targeting controls), at a low multiplicity of infection (MOI) of 0.3. The transduced cells were selected with 2 µg mL^−1^ of puromycin for 7 days to generate a mutant cell pool, which was then treated with DMSO or erdafitinib (100 µmol L^−1^) for 4 days. At least 3 × 10^7^ cells were harvested for gDNA extraction, ensuring over 400× coverage of the GeCKO v2 library. The sgRNA sequences were amplified using NEBNext High‐Fidelity 2X PCR Master Mix (NEB, M0541) and subjected to massive parallel amplicon sequencing carried out by Novogene Technology (Beijing, China). The sgRNA read count and hits calling were analyzed by MAGeCK v0.5.7 algorithm.^[^ ^46^ ^]^ The Crispr screening results have been uploaded to the Gene Expression Omnibus (GEO) repository, and the accession number is GSE276232.

### CRISPR/Cas9 Knockout

To generate COPA‐KO cell lines, COPA‐specific single‐guide RNA (sgRNA) was cloned into the lentiCRISPR v2 plasmid (Addgene Plasmid #52 961). HEK‐293T cells in a 10‐cm dish were transfected with 6 µg lentiCRISPR v2 vector containing COPA‐specific sgRNA and lentiviral packaging plasmids psPAX2 (2 µg), pMD2.G (1 µg) at a confluence of about 90%. The medium was replaced by 10 mL fresh DMEM (Gibco, USA) medium containing 10% FBS 6 h later. 48 h after transfection, cell medium containing lentivirus was harvested and filtered through a 0.45‐µm filter. MGH‐U3 and SW780 cells were then infected by the lentivirus at 80% confluence, followed by selection with 1µg/mL puromycin (Invitrogen, USA) for 1 week. Survival cells were seeded into 96‐well plates to obtain single cell‐derived clones. Knockout efficiency of COPA in single‐cell‐derived clones was verified by western blotting with the anti‐COPA antibody (A19651, ABclonal). The information on all sgRNA sequences used is listed in Table S1 (Supporting Information).

### RNA Preparation and qRT‐PCR

Total RNA from cell samples was extracted by RNeasy mini kit (Qiagen, Germany) following the manufacturer's instructions. cDNA was synthesized using HiScript III RT SuperMix (Vazyme, China). The quantitative real‐time polymerase chain reaction (qRT‐PCR) analyses were performed using SYBR Green Master Mix (Vazyme, China). The primers were listed in Table S1 (Supporting Information). The results were analyzed with the Step OnePlus Real‐Time PCR System (Applied Biosystems, USA). The 2−△△Ct method was used to quantify the transcript levels.

### Vector Construction and Cell Transfection

To construct COPA, LRPPRC, and ID3 overexpression plasmids, human COPA, LRPPRC, and ID3 cDNAs were synthesized by TSINGKE (Wuhan, China) and cloned into pcDNA3.1‐3 × Flag‐C vector. Truncations of COPA and LRPPRC were amplified with primers (Table S1, Supporting Information), and subcloned into pcDNA3.1‐3 × Flag‐C vector. Oligonucleotides encoding short hairpin RNAs (shRNAs) targeting LRPPRC, ID3, p21, and p16 (Table S1, Supporting Information) were synthesized by TSINGKE (Wuhan, China) and were cloned into pLKO.1 vector (Sigma–Aldrich, USA). Transfection was carried out using Lipofectamine 2000 (Life Technologies) according to the manufacturer's instructions. The transfected cells were then, respectively, selected with neomycin (Sigma–Aldrich, USA) or puromycin (Invitrogen, USA). Empty vector and scramble shRNA (sh‐scb) were applied as controls.

### Western Blotting

Cell lines were collected and lysed in RIPA buffer (Invitrogen, USA) supplemented with protease inhibitor cocktail (MCE, China). The concentration of total protein was measured by BCA protein assay kit (HYcezmbio, China). The protein samples were subjected to SDS‐PAGE and then transferred to a PVDF membrane, followed by blocking with 5% nonfat milk. Membranes were incubated with primary antibodies overnight at 4 °C on a shaker and incubated with HRP‐conjugated secondary antibodies for 1 h at room temperature before visualizing bands using enhanced chemiluminescence (G2020, Servicebio, China). The following antibodies were used: primary antibodies against GAPDH (60004‐1‐Ig, Proteintech), Tubulin (11224‐1‐AP, Proteintech), H3 (17168‐1‐AP, Proteintech), COPA (A19651, ABclonal), COPG (12393‐1‐AP, Proteintech), COPB1 (A10485, ABclonal), COPB2 (A22381, ABclonal), LRPPRC (21175‐1‐AP, Proteintech), ID3 (A5375, ABclonal), p21 (10355‐1‐AP, Proteintech), p16 (10883‐1‐AP, Proteintech), Flag (ab205606, Abcam); HRP‐conjugated secondary goat anti‐mouse (SA00001‐1, Proteintech), or goat anti‐rabbit (SA00001‐2, Proteintech) antibodies.

### Nuclear and Cytoplasmic Extraction

Nuclear and cytoplasmic fractions were isolated by Nuclear and Cytoplasmic Protein Extraction Kit (Beyotime, China) following the manufacturer's instructions. Briefly, cells were lysed in cytoplasmic buffer on ice for 15 min. After centrifugation at 16 000 × g for 5 min at 4 °C, the supernatant was collected as cytoplasmic protein. Then, the pellet was lysed in nuclear buffer on ice for 30 min. After centrifugation at 16000 × g for 10 min at 4 °C, the supernatant was collected as nuclear protein.

### Immunofluorescence Analysis

MGH‐U3 and SW780 cells grown on confocal dishes were fixed with 4% paraformaldehyde for 30 min and permeabilized with 0.1% Triton X‐100 for 10 min. Then cells were blocked with 1% BSA for an hour at room temperature before being treated with antibody specific for COPA (A19651, ABclonal), LRPPRC (21175‐1‐AP, Proteintech), and GM130 (11308‐1‐AP, Proteintech) at 4 °C overnight. The next day, the dishes were washed with PBS and then incubated with the corresponding secondary antibody for 1 h at room temperature, followed by sealing with DAPI. The images were captured using a Nikon A1Si Laser Scanning Confocal Microscope (Nikon Instruments Inc, Japan).

### CCK8 Assay

Cell viability was detected by CCK‐8 assay (HYcezmbio, China) following the manufacturer's instructions. Cells were cultured in 96‐well plates of 3 × 10^3^ per well and cultured in medium with DMSO or erdafitinib for different time periods respectively (0, 24, 48, 72, and 96 h). Then, CCK‐8 solution (10 µl) was added into each well and incubated at 37 °C for 2 h. The absorbance at 450 nm was measured using an automatic microplate reader (Synergy4; BioTek, Winooski, VT, USA). The proliferation index was defined as the ratio of the sample's OD value at each time point to that at 0 h.

### EdU Assay

Bladder cancer cells were seeded in 96‐well plates at a density of 3 × 10^4^ cells per well with or without erdafitinib treatment. The EdU assay was performed using the 5‐ethynyl‐2′‐deoxyuridine assay (EdU; EdU Cell Proliferation Kit with DAB, Beyotime, China) according to the protocol described. EdU (100 µmol L^−1^) was added to the medium and incubated with the cells for 2 h. The images were captured using Olympus FSX100 microscope (Olympus, Tokyo, Japan). The ratio of EdU‐stained cells to Hoechst‐stained cells was calculated to evaluate cell proliferation.

### Colony Formation Assay

MGH‐U3 and SW780 cells were cultured in 6‐well plates at a density of 800–1000 cells per well with or without erdafitinib treatment. Plates were incubated at 37 °C in 5% CO^2^ for 2–3 weeks, and colonies containing more than 50 cells were scored. Cell colonies were fixed with 4% paraformaldehyde for 30 min and stained with 0.1% crystal violet (Sigma–Aldrich, USA) for a further 30 min at room temperature.

### Flow Cytometry Assay for the Cell Apoptosis

For the apoptosis assay, cells were seeded into a six‐well plate with or without erdafitinib treatment. The cell apoptosis assay was determined according to the manual of FITC Annexin V Apoptosis Detection Kit I (BD Biosciences). Data were analyzed using FlowJo software (FlowJo).

### Tumor Xenograft Model

All animal experiments were approved by the Animal Care Committee of Tongji Medical College, and the IACUC number is 3897. Four‐week‐old female BALB/c nude mice were chosen for tumor xenograft experiments. Cells were injected subcutaneously into the dorsal flanks of mice (3 × 10^6^ cells per mouse). Tumor dimensions were quantified every week using a caliper and calculated according to the following formula: a^2^ × b × 0.52, where a is the smallest diameter and b is the diameter perpendicular to a. At the conclusion of the experiment, mice were euthanized, and tumors were excised and weighed. Erdafitinib or DMSO was administered by oral gavage at the dose of 20 mg kg^−1^ once daily from 1 week post‐injection to the conclusion of the experiment. All animal experiments were conducted following the NIH Guidelines for the Care and Use of Laboratory Animals.

### Histology and Immunohistochemistry (IHC)

Tumor tissues were fixed in 4% paraformaldehyde and embedded in paraffin. Immunohistochemistry examination was conducted using Ki‐67, p16, and p21 antibodies at Wuhan Powerful Biology.

### Luciferase Reporter Assays

The p16 and p21 promoter reporter vector (pGL3‐Basic vector) and ID3 3′‐UTR (WT and Mut) reporter vector (pmirGLO vector) were designed and synthesized by TSINGKE (Wuhan, China). The p16 and p21 promoter reporter were transiently transfected with Renilla control plasmid, in conjunction with ID3 overexpression/knockdown vectors. The ID3 3′‐UTR reporter was transiently transfected with LRPPRC overexpression/knockdown vectors. The Dual Luciferase Reporter Gene Assay Kit (Beyotime, China) was used to measure the luciferase activities following the manufacturer's instructions.

### Coimmunoprecipitation

In brief, 2 × 10^7^ cells were lysed in 2 mL Co‐IP buffer (20 mM Tris‐HCl, pH 7.5, 150 mM NaCl, 1 mM EDTA, and 0.5% NP‐40) supplemented with protease inhibitor cocktail (MCE, China) for 1 h on ice. A total of 5% of the cell lysate was used as input, while the remaining was incubated with protein A/G beads (MCE, China) for 2 h at room temperature and washed twice with PBST. Then, the mixture was divided equally into two portions and incubated with 4 µg of either the target or IgG antibody overnight at 4 °C. The complexes were washed twice with PBST, and all liquid was removed. The proteins were then removed from the beads with loading buffer under standard denaturing conditions. Purified proteins were detected by western blotting. The following secondary antibodies were used for immunoblotting: HRP‐goat anti‐mouse IgG heavy chain specific (AS064, ABclonal), anti‐mouse IgG light chain specific (AS062, ABclonal).

### Silver Staining and Mass Spectrometry Analysis

Silver staining was conducted following the protocol described in the PAGE Gel Silver Staining Kit (Solarbio, Beijing, China). Mass spectrometry analysis was performed by APTBIO (Shanghai, China). Protein identification and quantification were performed using Proteome Discoverer software (version 1.4; Thermo Fisher Scientific, USA).

### Cycloheximide Chase Assay

Cycloheximide was added to the culture medium at a final concentration of 20 µM. Cell lysates were collected at 0, 2, 4, 6, and 8 h after cycloheximide treatment and immunoblotting was performed to evaluate the corresponding protein levels.

### RNA Immunoprecipitation (RIP)

The RIP assay was conducted following the instructions provided in the Magna RIPTM RNA‐Binding Protein Immunoprecipitation Kit (Millipore). In brief, approximately 1 × 10^7^ cells in a 15‐cm dish were harvested and lysed to extract total protein before being cross‐linked at 254 nm (200 J cm^−2^) by UV light with ice for 1 min. Total protein was immunoprecipitated with antibodies against LRPPRC (21175‐1‐AP, Proteintech) and rabbit IgG (ab172730, Abcam). Following proteinase K treatment, input and co‐immunoprecipitated RNAs were extracted using RNeasy Mini kit (QIAGEN, Germany) according to the manufacturer's instructions and analyzed by RT‐qPCR.

### MeRIP‐qPCR

The MeRIP assay was performed using an N6‐Methylated RNA Immunoprecipitation (MeRIP) Kit (Bes5203, BersinBio, China) according to the guidelines. Briefly, total RNA was fragmented to approximately 300 nt using fragmentation buffer at 94 °C for 2 min, and ethylenediaminetetraacetic acid (EDTA) was added to stop the reaction. 50 µL of the 950 µL fragmented RNA sample was saved as an input control, and the remaining fragmented RNA was equally divided for immunoprecipitation with 5 µg of m6A antibody (68055‐1‐Ig, Proteintech) or IgG (AC011, ABclonal) at 4 °C overnight. The prepared Protein A/G magnetic beads were incubated with IP Mix buffer at 4 °C for 3 h, then the beads were digested with Proteinase K at 55 °C for 45 min. The IP‐fragmented RNA was extracted with phenol‐chloroform‐isoamyl alcohol. The m6A status of ID3 was further measured by RT‐qPCR using the m6A peak primers in Table S1 (Supporting Information).

### Actinomycin D Treatment and RNA Stability Assay for RNA Lifetime

For the purpose of conducting an actinomycin D treatment, cells were plated in six‐well plates. Following a 24‐h period, during which the cells reached a confluency level of up to 60%, they were treated with 5 µg mL^−1^ actinomycin D or DMSO, and samples were collected at the indicated time points. The total RNA was extracted using TRIzol reagent (Invitrogen) following the manufacturer's instructions and analyzed by qRT‐PCR. The turnover rate and half‐life of RNA were estimated according to a previously published paper.^[^ ^47^ ^]^

### RNA‐seq

Total RNA from cell samples was extracted by RNeasy mini kit (Qiagen, Germany) following the manufacturer's instructions. The transcriptome profiles of sg‐NC and COPA‐KO cells with DMSO or erdafitinib treatment were determined by RNA‐seq (Novogene, Tianjin, China). Differentially expressed RNAs of P‐value top 800 were used for further analysis. The RNA‐seq data have been uploaded to the GEO repository, and the accession number is GSE278672.

### Statistics Analysis

The data was presented as Mean ± SD. Statistical analysis was conducted with GraphPad Prism 8.0 software, utilizing student t‐test to compare differences between groups. P < 0.05 was considered statistically significant.

## Conflict of Interest

The authors declare no conflict of interest.

## Author Contributions

H.Z., X.G., and Y.J. contributed equally to this work. G.J., H.Z., and C.H. contributed to the design of this study. H.Z., Y.J., and Y.Y. performed main experiments and analyzed data. X.G., J.S., and L.W. analyzed bioinformatics data. M.W., Y.J., and X.X. performed statistical analyses. H.Z., H.Z., Y.J., and G.J. contributed to writing, review, and/or revision of the manuscript. All authors read and approved the final manuscript.

## Supporting information



Supporting Information

Supplemental Table 1

Supplemental Table 2

Supplemental Table 3

Supplemental Table 4

Supplemental Table 5

## Data Availability

The data that support the findings of this study are openly available in GEO at https://www.ncbi.nlm.nih.gov/geo/query/acc.cgi?acc=GSE276232, reference number 276232.
