# Supplementary material for: Targeting COPA to Enhance Erdafitinib Sensitivity in FGFR‐Altered Bladder Cancer
Source: Adv Sci (Weinh). 2025 Mar 20;12(18):2413209. doi: 10.1002/advs.202413209 (PMC12079435; doi:10.1002/advs.202413209)
Supplement: Supplementary file 1 — Supporting Information [file ADVS-12-2413209-s004.docx]

**Targeting COPA to Enhance Erdafitinib Sensitivity in FGFR-Altered Bladder Cancer**

*Huayuan Zhao^#^, Xincheng Gao^#^, Yangkai Jiang^#^, Yanchao Yu, Liang Wang, Jiayin Sun, Miao Wang, Xing Xiong, Chao Huang^*^, Hui Zhang^*^, Guosong Jiang^*^*


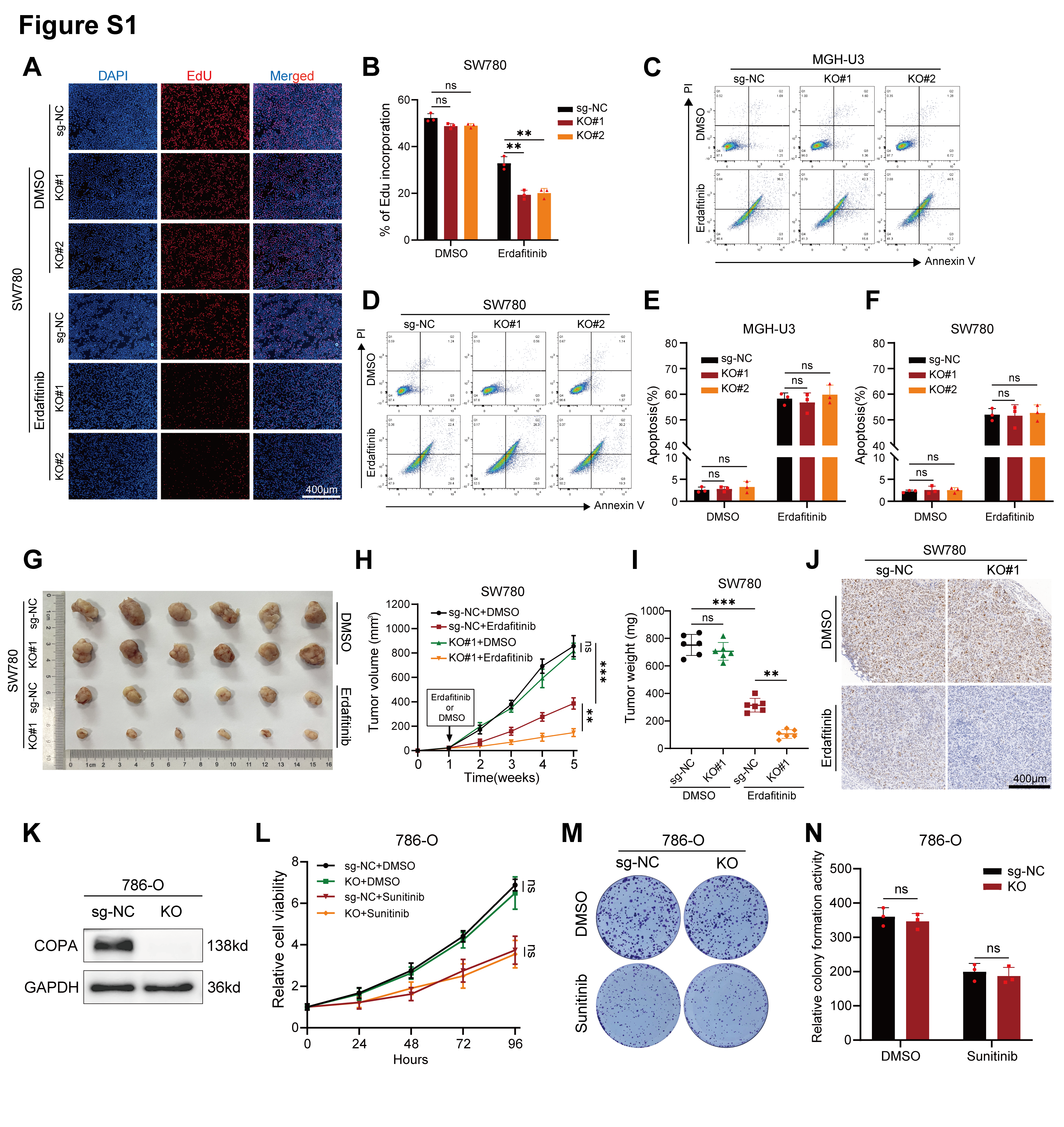


**Supplementary Figure 1. Knockout of COPA inhibits the proliferation of bladder cancer cells in vitro and in vivo.**

**(A, B)** EdU assay showed the proliferation of sg-NC and COPA-KO SW780 cells treated with DMSO or erdafitinib (*n* *=* 3). Scale bar, 400 μm.

**(C-F)** Flow cytometry assay revealed the rate of apoptosis in MGH-U3 and SW780 cells (before and after COPA knockout) treated with DMSO or erdafitinib (*n =* 3).

**(G-I)** Representative (G), in vivo growth curve (H), and weight at the endpoints (I) of xenograft tumors formed by subcutaneous injection of sg-NC and COPA-KO SW780 cells into the right flanks of nude mice treated with DMSO or erdafitinib (20 mg/kg) (5 × 10^6^ cells per mouse; *n =* 6 for each group).

**(J)** IHC staining of Ki67 on sg-NC and COPA-KO SW780 xenografts treated as in (H). Scale bar, 400 μm.

**(K)** The efficiency of COPA knockout in 786-O cells was detected by western blotting. GAPDH was used as internal control.

**(L)** CCK-8 assay revealed the cell viability of 786-O cells (before and after COPA knockout) treated with DMSO or sunitinib (*n =* 3).

**(M, N)** Colony formation assay was performed in 786-O cells (before and after COPA knockout) treated with DMSO or sunitinib (*n =* 3).

Data are represented as mean ± SD. ns indicates not significant. **P < 0.01; ***P < 0.001 (Student t test).


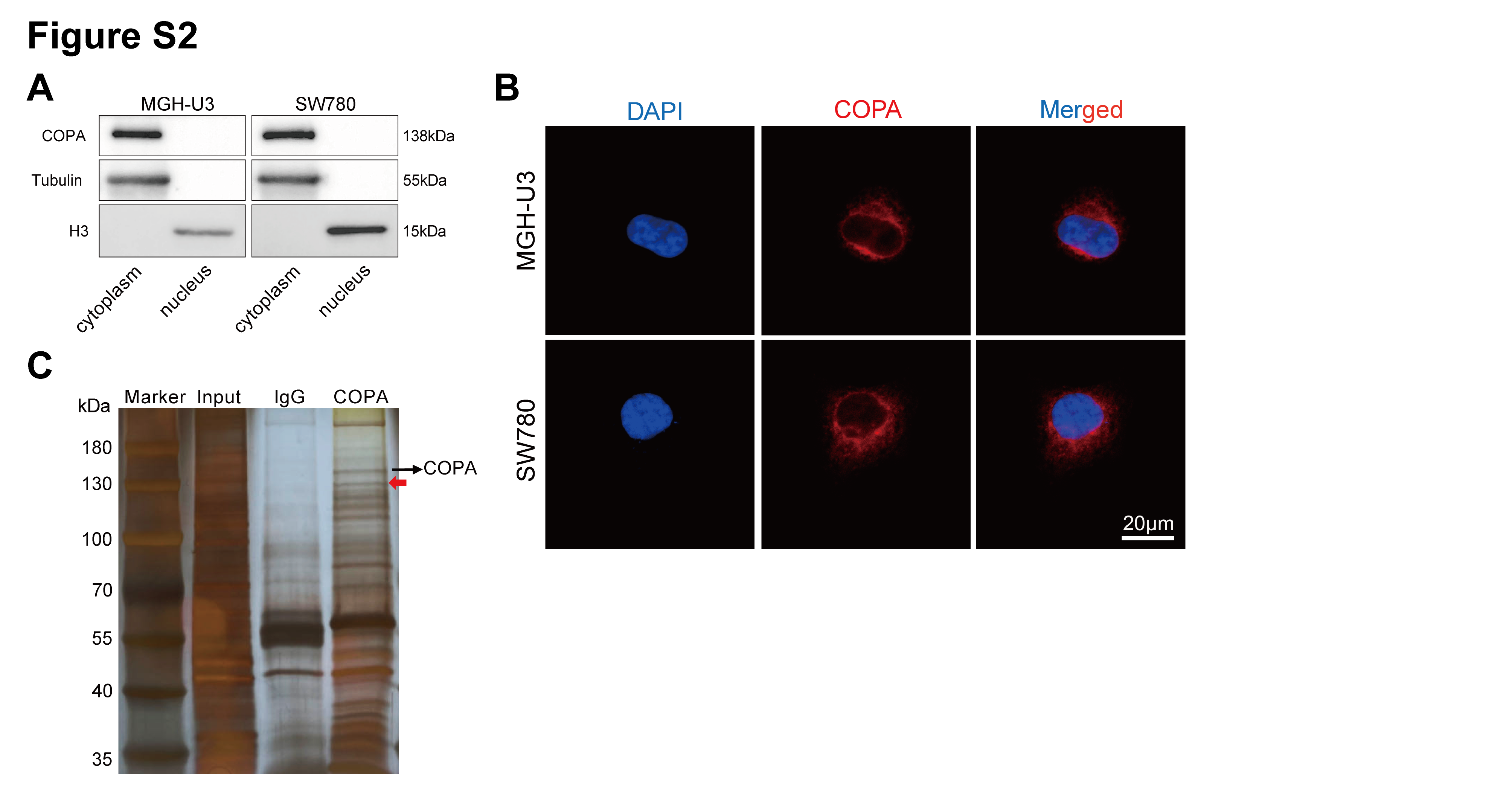


**Supplementary Figure 2. COPA is mainly located in the cytoplasm of MGH-U3 and SW780 cells**

**(A)** Western blotting indicated the distribution of COPA in MGH-U3 and SW780 cells; H3 and Tubulin were applied as positive controls in the nucleus and cytoplasm, respectively.

**(B)** Immunofluorescence staining assay showed the distribution of COPA (red) in MGH-U3 and SW780 cells; nuclei (blue) were stained with DAPI. Scale bar, 20 μm.

**(C)** Silver staining showed the proteins pulled down by COPA from the lysates of SW780 cells. Red arrow indicated the major differential band precipitated.


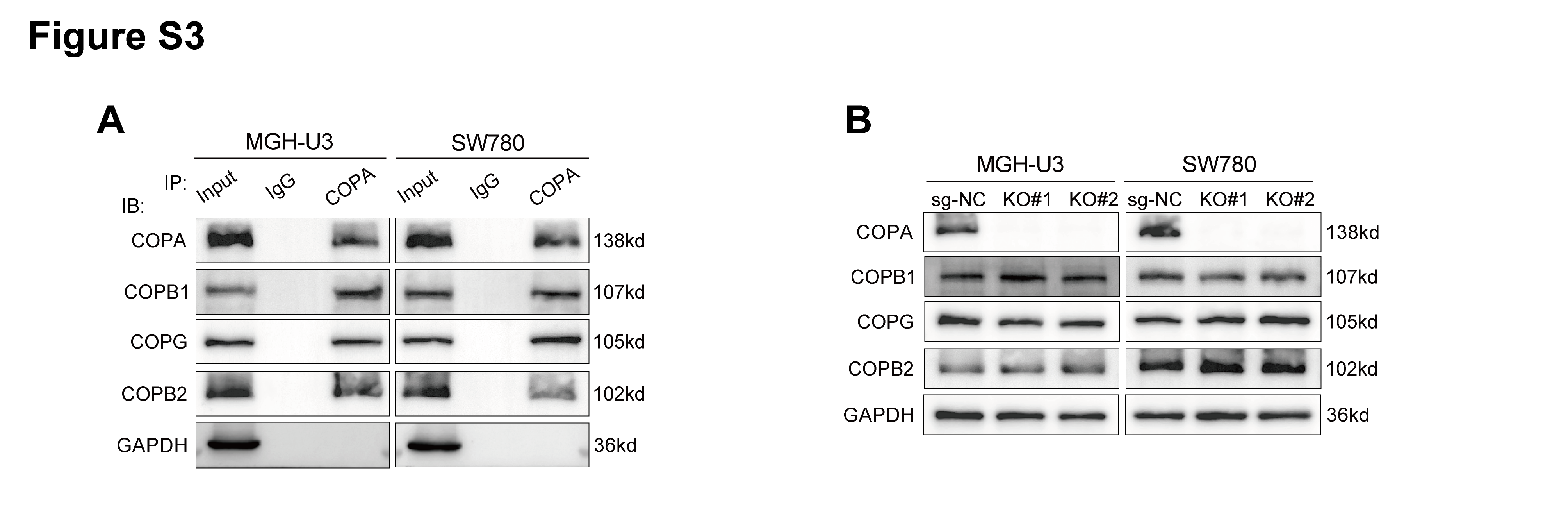


**Supplementary Figure 3. COPA interacts with other COPI subunits.**

**(A)** Co-IP assay using antibody specific for COPA showed the interaction between COPA and other COPI subunits in MGH-U3 and SW780 cells. The precipitate was subjected to western blotting with the indicated antibodies.

**(B)** The expression of residual COPI subunits in MGH-U3 and SW780 cells before and after COPA knockout was detected by western blotting. GAPDH was used as internal control.


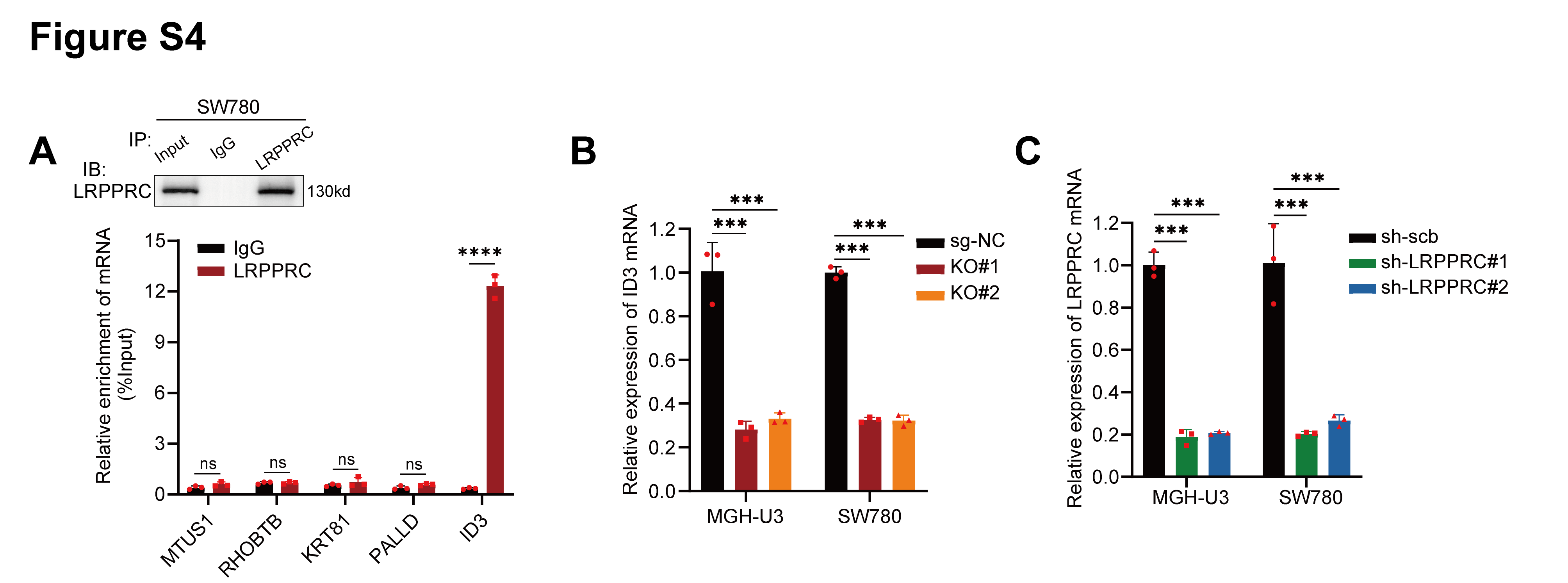


**Supplementary Figure 4. ID3 is the target of LRPPRC in the regulation of erdafitinib sensitivity**

**(A)** RIP assays in SW780 cells using LRPPRC and IgG antibody. The precipitate was subjected to western blotting with the antibody against LRPPRC. The LRPPRC-enriched mRNAs relative to the IgG-enriched value was calculated by qRT-PCR (*n =* 3).

**(B)** The expression of ID3 in MGH-U3 and SW780 cells before and after COPA knockout was detected by qRT-PCR (*n =* 3).

**(C)** The expression of LRPPRC was detected by qRT-PCR in MGH-U3 and SW780 cells transfected with scramble, sh-LRPPRC#1 or sh-LRPPRC#2 (*n =* 3).

Data are represented as mean ± SD. ns indicates not significant. ***P < 0.001; ****P < 0.0001 (Student t test).


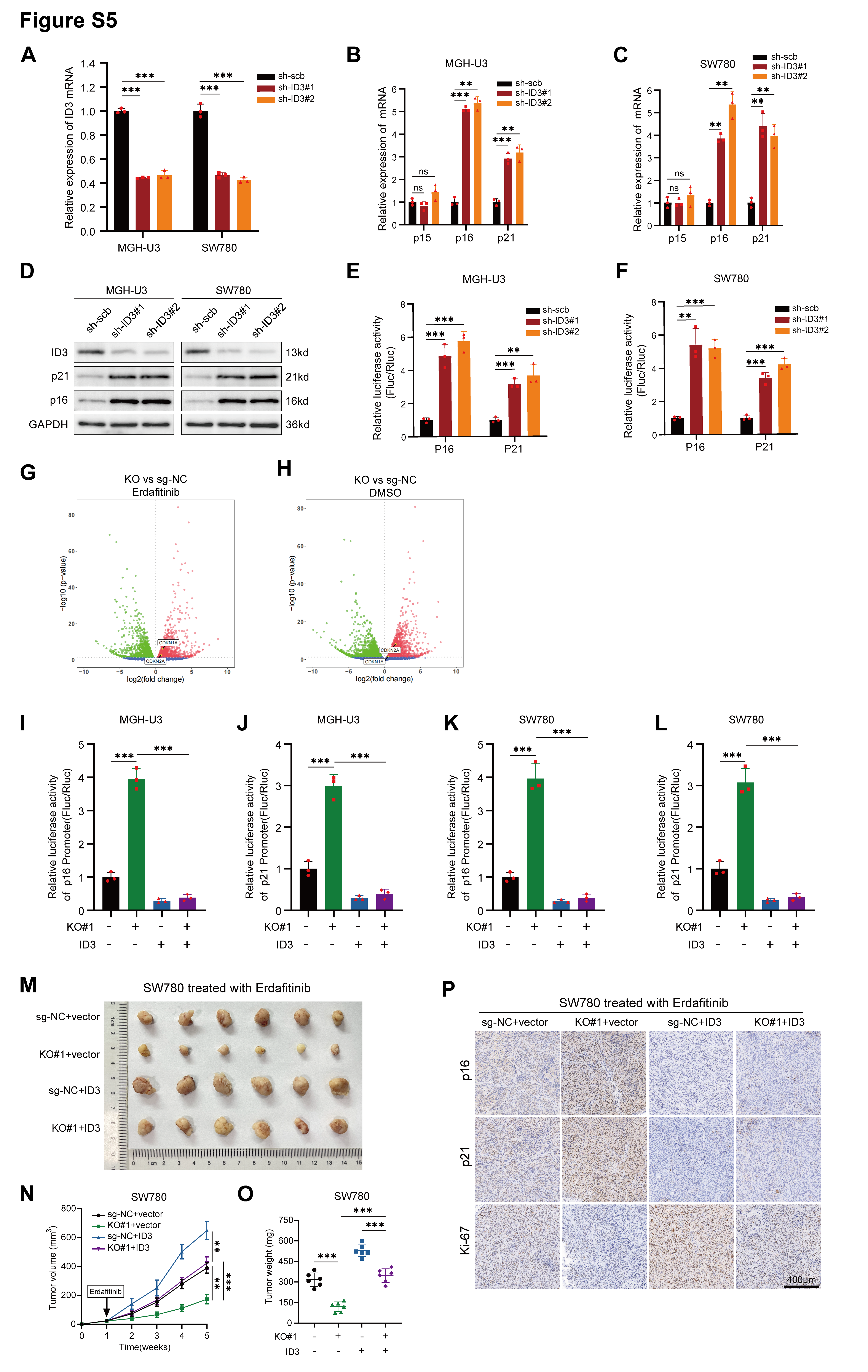


**Supplementary Figure 5. ID3 mediates the regulation of COPA on the transcriptional activity of p16 and p21, as well as on the sensitivity to erdafitinib.**

**(A)** The expression of ID3 in MGH-U3 and SW780 cells transfected with scramble, sh-ID3#1 or sh-ID3#2 was detected by qRT-PCR (*n =* 3).

**(B, C)** The expression of p15, p16 and p21 in MGH-U3 (B) and SW780 (C) cells transfected with scramble, sh-ID3#1 or sh-ID3#2 was detected by qRT-PCR (*n =* 3).

**(D)** Western blotting with the indicated antibodies in MGH-U3 and SW780 cells transfected with scramble, sh-ID3#1 or sh-ID3#2. GAPDH was used as internal control.

**(E, F)** The relative luciferase activity of p16 and p21 promoter in MGH-U3 (E) and SW780 (F) cells transfected with scramble, sh-ID3#1 or sh-ID3#2 (*n =* 3).

**(G, H)** The volcano map illustrated the differentially expressed mRNAs in sg-NC and COPA-KO MGH-U3 cells, with CDKN2A (p16) and CDKN1A (p21) annotated.

**(I-L)** The relative luciferase activity of p16 and p21 promoter in MGH-U3 (I, J) and SW780 (K, L) cells (before and after COPA knockout) transfected with vector or ID3 (*n =* 3).

**(M-O)** Representative (M), in vivo growth curve (N), and weight at the endpoints (O) of xenograft tumors formed by subcutaneous injection of sg-NC and COPA-KO SW780 cells transfected with vector or ID3 into the right flanks of nude mice treated with erdafitinib (20 mg/kg) (5 × 10^6^ cells per mouse; *n =* 6 for each group).

**(P)** IHC staining of Ki-67, p16 and p21 on sg-NC and COPA-KO SW780 xenografts treated as in (N). Scale bar, 400 μm.

Data are represented as mean ± SD. ns indicates not significant. **P < 0.01; ***P < 0.001 (Student t test).


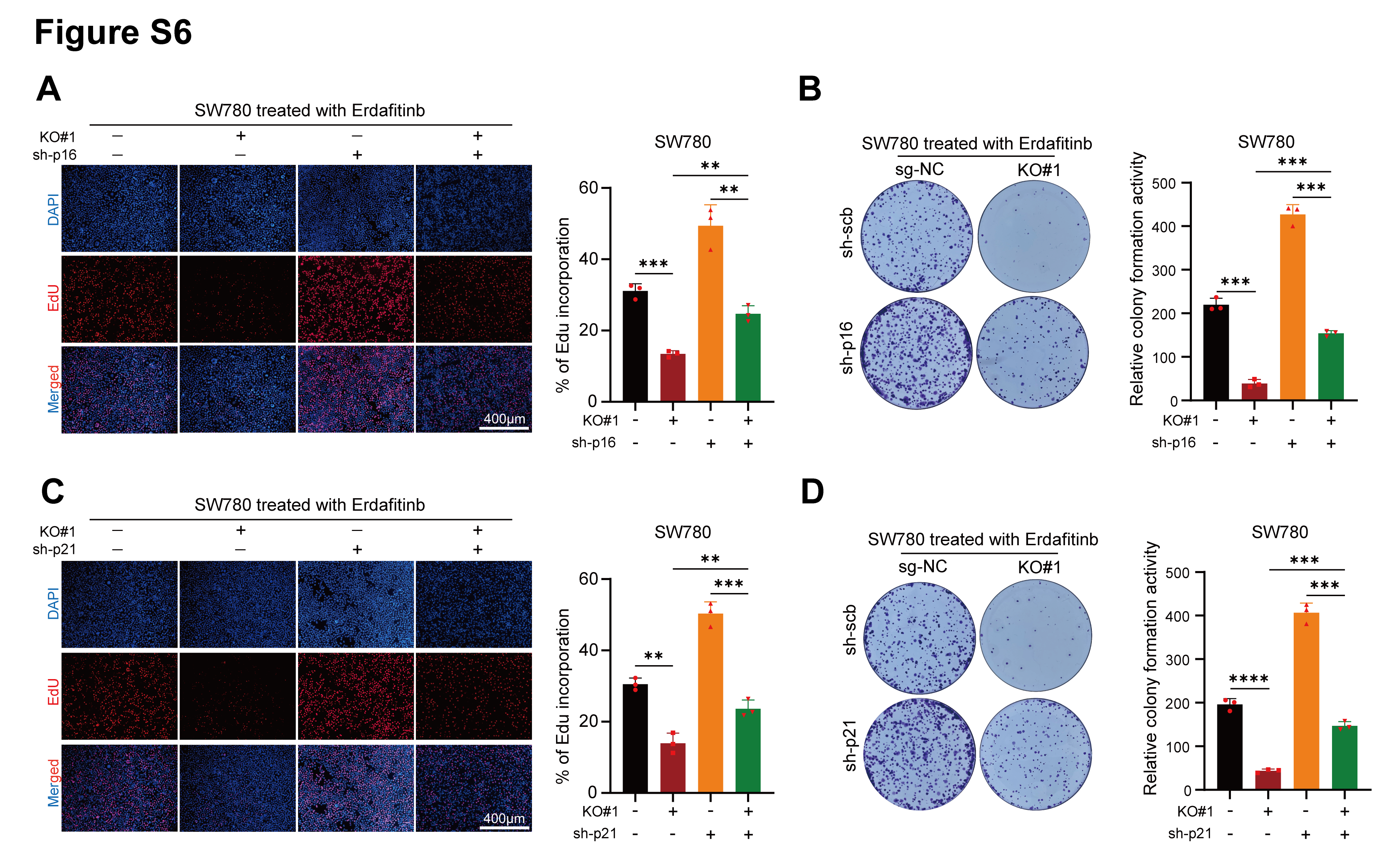


**Supplementary Figure 6. p16 and p21 mediate the regulation of COPA on the sensitivity to erdafitinib.**

**(A)** EdU assay showed the proliferation of sg-NC and COPA-KO SW780 cells transfected with scramble or sh-p16 treated with erdafitinib (*n =* 3). Scale bar, 400 μm.

**(B)** Colony formation assay was performed in sg-NC and COPA-KO SW780 cells transfected with scramble or sh-p16 treated with erdafitinib (*n =* 3).

**(C)** EdU assay showed the proliferation of sg-NC and COPA-KO SW780 cells transfected with scramble or sh-p21 treated with erdafitinib (*n =* 3). Scale bar, 400 μm.

**(D)** Colony formation assay was performed in sg-NC and COPA-KO SW780 cells transfected with scramble or sh-p21 treated with erdafitinib (*n =* 3).

Data are represented as mean ± SD. **P < 0.01; ***P < 0.001; ****P < 0.0001 (Student t test).
